# Role and Efficacy of Intraoperative Evaluation of Resection Adequacy in Conservative Breast Surgery

**DOI:** 10.5402/2011/247385

**Published:** 2011-04-28

**Authors:** G. Canavese, G. Ciccarelli, L. Garretti, A. Ponti, R. Bussone, R. Giani, A. Ala, E. Berardengo

**Affiliations:** ^1^Dipartimento di Patologia, Ospedale San Giovanni Antica Sede, A.S.O. San Giovanni Battista Molinette, Via Cavour 31 10126 Torino, Italy; ^2^Dipartimento di Radiologia, Ospedale San Giovanni Antica Sede, A.S.O. San Giovanni Battista Molinette, 10126 Torino, Italy; ^3^Centro di Riferimento per l'Epidemiologia e la Prevenzione Oncologica in Piemonte (CPO Piemonte), 10126 Turin, Italy; ^4^Dipartimento di Chirurgia del Seno, Ospedale San Giovanni Antica Sede, A.S.O. San Giovanni Battista Molinette, 10126 Torino, Italy

## Abstract

In the present study we considered the histology of 51 patients who have undergone breast conservative surgery and the related 54 re-excisions that were performed in the same surgical procedure or in delayed procedures, in order to evaluate the role of intraoperative re-excisions in completing tumor removal. In 13% of the cases the re excision obtained the resection of the target lesion. In this study, the occurrence of residual neoplastic lesions in intraoperative re-excisions (24%) is lower than in delayed re-excisions (62%; *P* = .03). The residual lesions that we could find with definitive histology of re excision specimens are related with lesions with ill defined profile. In 77% of the cases of re excision with tumoral residual the lesion was close to the new resection margin, thus the re-excisions couldn't achieve an adequate ablation of the neoplasm. Invasive or preinvasive nature of the main lesion resected for each case and the approach to the evaluation of the first resection specimen adequacy (surgical or radiological) don't affect the rate of tumoral residual in intraoperative re-excisions. In conclusion, our data are consistent with a low efficacy of intraoperative re excision in obtaining a complete removal of the tumor; intraoperative radiologic evaluation of the first resection specimen is however imperative in defining the effective removal of the target lesion.

## 1. Introduction

Breast oncologic surgery is now widely focussed in conservative treatment with tissue sparing in order to obtain satisfying cosmetic results besides an adequate surgical resection [1–3]. Obviously, resection borders are becoming considerably closer to the neoplastic lesions; hence, tumor-free margins are hardly achieved in nonpalpable lesions, such as small lesions, lesions with ill-defined profile, and microcalcifications areas. A large number of studies proved that the condition of the resection margins represents a significant risk factor of recurrences in women who underwent breast conservative surgery, together with tumor size and tumor grading (see recent reviews from Singletary and Huston) [4, 5]. It was claimed that margin status represents an independent risk factor in distant metastasis development and overall survival. [6–8]. On the other hand, the worth of close or involved margins in predicting the presence of neoplastic residual in the area next to the surgical bed is still debated: a recent paper reported that 21% of a series of re-excised tumors with negative margins contained residual tumors [9]. Unfortunately, some inconsistencies in reported data depend on the fact that a large consensus about the definition of free surgical resection margins has still to be reached.

In recent years, the role of the intraoperative evaluation of surgical specimen in resection margin control was assessed, but the literature is still limited and dissimilar. 

A group of recent papers on series of conservative resections for breast malignancies proved that the intraoperative examination of resection specimens is useful in decreasing the rate of second procedures, employing gross examination techniques [10], specimen radiogram [11], or a combination of them [12]. Another paper proved the efficacy of gross margin assessment combined with radiography in skin sparing mastectomies in reducing excision rate in breast conserving surgery for carcinoma in situ [13]. 

Assuming that the re-excision procedures are useful tools in obtaining an adequate removal of the neoplastic lesions, thus reducing the risk of persistence of neoplastic foci in residual parenchyma, we believe it could be of interest to make an attempt at quantifying the amount of tumoral mass that lies beyond a margin that is considered close according to the ordinary criteria of intraoperative evaluation (mammography of the operative specimen, clinical examination of surgical bed specimen, and frozen sections examination). 

The literature about this topic is still somewhat limited and is mainly based on delayed excisions: two recent works [14, 15] analyzed 23 and 26 delayed re-excisions after positive surgical margins at definitive histology of primary surgery. The rate of re-excision with residual tumors was, respectively, 48% and 65%, and one of these studies stated that none of the examined risk factors was statistically related with the occurrence of residual tumor in re-excision specimen. 

The present survey is based on the examination of histological material from a consecutive series of conservative resections that comprehended re-excision procedures, both in the context of the same session and in delayed surgical treatments. The main goal of this study is the assessment of the extension and of the morphological characteristics of the tumoral residuals in re-excision specimens to define both the role of the re-excisions (and mainly of the limited re-excisions) as a tool to obtain the complete removal of neoplastic lesions in conservative breast surgery and the potential employment of other therapeutic options in obtaining an adequate removal of the target area. 

As a consequent achievement, we planned to weigh up some of the issues that could be related with the occurrence of tumoral residuals, as the invasive or preinvasive nature of the lesion, the method of evaluation of the first resection adequacy, and the time occurring between the first resection and the re-excision.

## 2. Materials and Methods

For the present study, we selected 51 patients consecutively treated in our institution with a preliminary conservative approach, subsequently extended with further (intraoperative or delayed) procedures. Since resection specimen radiogram is considered the most reliable technique of margin status evaluation in our structure, all the first resection specimens were conveyed to the radiology department for intraoperative evaluation, and most of the re-excisions were based on the radiologist's advice. 

Considering the main lesions reported in final histology for each patient, 28 infiltrating carcinomas (55%, 4 multifocal), 19 in situ carcinomas (37%), and 4 (8%) benign lesions were resected. All the benign lesions were reported at final histology as sclerosing adenosis.

The main histological characteristics of infiltrating and in situ carcinomas are resumed in Table 1.

112 surgical specimens from 54 procedures (first resections and subsequent re-excisions) were evaluated in this study.

### 2.1. Specimen Radiography Protocol

Resection specimens were examined in radiology department using two standard projections. The presence of the target lesion in the radiograms was first ascertained; the margins were judged close to the target lesion when the lesion was eccentrically placed in the same mammograms.

### 2.2. Histological Protocol

Space-oriented specimens were examined for the final histology. In the initial resections, the surgical margins were marked with one or two different colour inks. In the re-excision specimens, the new resection margins were inked. Consecutive sections of the area of the neoplasm closest to the surgical margins were obtained in large specimens while the whole tissue was processed in smaller specimens or, in the cases in which the lesion's shape was not clearly identifiable, on macroscopic examination. All macroscopically significant areas were processed. 

Resection margin was considered close when the distance from the lesions was equal to or smaller than 2 mm.

Histological slices were reviewed by two different pathologists.

### 2.3. Statistical Evaluation

*X^2^* test was used for statistical evaluations cited in the results section.

## 3. Results

52 patients were enrolled for this study, with 54 related re-excisions.

We divided the re-excisions in accordance with the time of re-excision and with the method of evaluation that defined the surgical procedure.

38 patients (74.5%) were re-excised intraoperatively: in 25, re-excision was supported by the radiological report of close margins after specimen mammogram and, in 13, direct re-excision was supported by clinical evidence of incomplete excisions during surgery; 3 patients of this group had delayed re-excisions performed after definitive histological report of close margins on first resection specimen.

The remaining 13 patients (25.5%) had no intraoperative extension, since the ordinary method of immediate evaluation of first resection provided suggestions for further procedures, and were extended subsequently (with 13 related specimens) because of evidence of close margin on histology.

**Figure 1:**
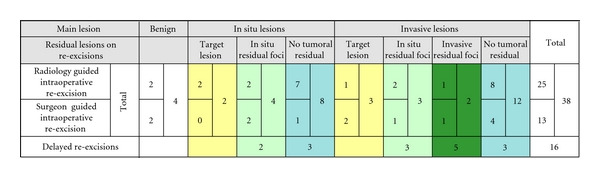
Residual lesions in re-excisions, ordered by main lesions detected at histology for any single case, and by suggestions for re-excision procedure.

The histological definitive diagnosis of the re-excisions specimens (considering the main lesion if the re-excisions consisted of more than one specimen) is reported in Figure 1, matched with the main lesion reported at final histology for each case.

### 3.1. Occurrence and Histological Features of Residual Lesions in Intraoperative and Delayed Re-excisions

The overall occurrence of residual neoplastic lesions in re-excisions selected for this study was 19 out of 54 (35%).

In the series of the intraoperative procedures concerning patient with neoplastic lesions, 5 (13%) were effective in removing the target lesion, 9 (24%) harboured residual neoplastic lesions, and 20 (52%) were reported as normal breast parenchyma or contained benign lesions at definitive histology. On the other hand, 4 intraoperative re-excisions (11%) were related to cases with definitive histological diagnosis of benign lesions. Among the 16 delayed re-excisions, 10 (62%) were effective in eradicating residual tumor.

The rate of residual lesions was compared in the two groups of intraoperative re-excisions (9/29 re-excisions, exclusive of the 5 re-excisions containing the target lesion and the 4 resections from patients with final diagnosis of benign lesion) and delayed re-excisions (10/16 re-excisions). The difference was statistically significant (*P* = .03) even considering the re-excision in the patients with final diagnosis of invasive carcinomas (28 re-excisions, 17 intraoperative, and 11 delayed; *P* = .01).

Considering the morphological features of the 9 intraoperative re-excisions which were effective in removing neoplastic residual, in 8 cases the residual lesions were scattered foci of in situ carcinomas: 4 re-excisions performed in cases with definitive diagnosis of in situ carcinoma contained residual foci of the lesion, 4 re-excisions performed in cases with main diagnosis of invasive carcinoma contained residual foci of peritumoral in situ in 3 cases, and a focus of microinvasion (2 mm) in an area of in situ carcinoma in another case. In 3 of the 8 excisions, the in situ carcinomas were poorly differentiated sec Holland.

Another re-excision harboured residual foci of a lobular carcinoma with invasive features.

In 7 cases (77%), the residual lesion was close to the new resection margin.

### 3.2. Invasive Preinvasive Features and Re-excision Efficacy

In order to assess the role of invasive features in the efficacy of re-excision, we evaluated the rate of residual lesions in re-excisions in the two groups of patients with final diagnosis of invasive carcinoma (13/28) and with final diagnosis of in situ carcinomas (6/17). We excluded re-excision from patients in which the re-excision led to the removal of the target lesion (5 re-excisions).

No statistical difference in the distribution of the neoplastic residual lesions between the two categories could be stated, both considering (*P* = .45) and ruling out (*P* = .45) the peritumoral in situ as residual lesion in resection for invasive lesions. 

Similar results were obtained considering only the intraoperative re-excisions (29 cases, *P* = .87 and *P* = .16, resp.).

### 3.3. Role of Resection Specimen Adequacy Evaluation in Intraoperative Re-excisions Efficacy

The role of the technique of intraoperative evaluation of the first resection in removing residual neoplastic lesion was assessed in the group of the 29 intraoperative re-excisions, using the same criteria of exclusion described in the preceding sections.

We matched the 20 radiology guided re-excisions and the 9 surgeon guided re-excisions. No statistical difference in the rate of neoplastic lesions was ascertained in the two groups (*P* = .10, *P* = .27, ruling out peritumoral in situ).

## 4. Discussion

Recent advances in breast surgery showed the efficacy of the conservative approach in surgical resection of breast carcinomas.

One of the main requirements in conservative resection is to obtain a complete removal of neoplastic lesions, usually verified with tumor-free resection margins at final histology. The goal of tumor-free margins is reached in recent surgical practice with intraoperative resection specimen examination, mostly with mammography and with specimen frozen section microscopy. In other cases, the purpose is obtained with delayed re-excision or radicalizations ensuing from pathological evidence of close margins at first resection.

The main purpose of this study is the evaluation of the efficacy of the immediate re-excision, performed after intraoperative evaluation of first re-excision specimen, in removing neoplastic residual. We believe it could be of interest to weigh the value of intraoperative re-excisions in obtaining the complete removal of the neoplastic mass, compared with other therapeutic options.

A preliminary statement is that a significant part of (13%) of the re-excisions leads to the resection of the target lesions, supporting the usefulness of this practice in breast oncologic surgery.

Considering the efficacy of intraoperative re-excisions in clearing residual parts of the main lesion, the histological definitive examination detected residual neoplasm in 24% of the cases. Interestingly, the rate of successful re-excision in this study is somewhat lower than the rate of occurrence in other studies available in the literature (see introduction) [9, 14–17]. This discrepancy could be explained with different settlement of close margins or a dissimilar evaluation of specimens at mammography. 

If we look at the morphology of tumoral residuals in limited intraoperative re-excisions that were confirmed as neoplastic lesions at definitive histology, mutifocal, ill-defined lesions (8 re-excisions with in situ foci, with one case of microinvasion, and one re-excision with lobular invasive carcinoma foci) were detected, and in the 77% of cases the re-excisions were ineffective in completing the removal of the neoplasm, as the residual lesions were close to the new resection margin. 

Our data show that invasive or preinvasive nature of target lesion does not affect the rate of neoplastic lesions in re-excisions: it could be expected that the oncological adequacy of conservative surgery is dependent on lesion profile and on detection capability at radiological and clinical examination, more than on evidences of invasion.

Postsurgical radiotherapy could be a good option in obtaining the local control of the residual lesion that we detected in our revision. A study on predictive factors of residual-positive re-excisions performed on 115 delayed resections [16] achieved the same statements. Another study [17] stated that recurrences in non-reexcised lumpectomies with negative and positive margins are not statistically different after radio chemotherapy.

According to our data, the efficacy of the intraoperative re-excisions (grouping together radiogram and clinical guided re-excisions) is lower than the efficacy of delayed surgery after histological examination of first resection specimen, both considering all the re-excisions with cases with final diagnosis of neoplastic lesion (excluding those which harboured the target lesion; *P* = .004) and limiting the analysis to cases with final diagnosis of invasive lesions. Placing these results in the background of the debate about the real implication of the finding of close margins, apart from the evaluation methods, our findings could not confirm the hypothesis of a higher rate of tumoral residual in immediate versus delayed re-excision for the absence of repair processes [18]; on the contrary, delayed re-excisions after histological evaluation of the first specimen seem to be more effective in removing tumoral tissue. 

Considering the evaluation method of the first resection adequacy, we could not ascertain any statistical difference between the efficacy of radiological evidence guided re-excisions and the surgeon's choice dependent re-excisions in completing tumoral excision. Probably, a larger number of cases are needed for a more accurate evaluation of these two techniques, but these results show that surgeon's evaluation of surgical bed has a pivotal role in removing neoplastic residual that are not evident in radiology. 

Concluding, this analysis suggests that intraoperative re-excisions are mandatory when the intraoperative examination doesn't confirm the presence of the target lesions in the first resection specimen, but is more questionable when the lesion is judged close to the resection margin. The lesions that were re-excised in the same surgical session were mainly ill-defined areas with foci of in situ carcinomas, and these findings suggest the employment of other therapeutic options, such as radiotherapy.

Considering the adequacy of the resections, it must be underlined that the practice of conservative breast surgery must now face the recent theory of the “sick lobe” which asserts that conservative breast surgery must obtain the complete resection of the whole lobe involved in neoplastic disease [19]. Following this theory, the resection of ill-defined neoplastic lesions such as in situ lesions, lobular invasive, and multifocal invasive carcinomas should be firstly planned in preoperative phase, with an accurate definition of the lesion's profile and with a careful evaluation of radiograms and other instrumental data (ultrasound, MR), integrated with cytological and microhistological presurgical sampling. On the other hand, the higher efficacy of the delayed re-excisions points out that the examination of the specimen with definitive histology is a more suitable procedure for establishing the morphological and biological characteristics of the lesion excised with the first excision and for planning further re-excisions.

## Figures and Tables

**Table 1:** Histological characteristics of the lesions.

In situ lesions					
Histological grading^(1)^				Maximum diameter range (cm)	4–19
G1	7				
G2	5				
G3	7			Mean diameter (cm)	7.57

Invasive lesions					

Staging^(2)^		Grading^(3)^		Histotype	
1mic	2	1	7	Ductal	17
1a	7	2	12	Ductal, main in situ	1
1b	6	3	9	Lobular	4
1c	11			Mixed, ductal, and lobular	4
2	2			Tubular	1
				Mucoid	1

^(1)^Holland R, Peterse JL, Millis RR, Eusebi V, Faverly D, van de Vijver MJ, Zafrani B. Ductal carcinoma in situ: a proposal for a new classification. Semin Diagn Pathol. 1994 Aug;11(3):167-80.

^(2)^TNM sixth edition Wiley Liss.

^(3)^Elston CW, Ellis IO. Pathological prognostic factors in breast cancer. I. The value of histological grade in breast cancer: experience from a large study with long-term follow-up. Histopathology. 1991 Nov;19(5):403-10.

## References

[1] Veronesi U, Salvadori B, Luini A (1990). Conservative treatment of early breast cancer. Long term results of 1232 cases treated with quadrantectomy, axillary dissection, and radiotherapy. *Annals of Surgery*.

[2] Veronesi U, Cascinelli N, Mariani L (2002). Twenty-year follow-up of a randomized study comparing breast-conserving surgery with radical mastectomy for early breast cancer. *The New England Journal of Medicine*.

[3] Zurrida S, Costa A, Luini A, Galimberti V, Sacchini V, Intra M (2001). The Veronesi quadrantectomy: an established procedure for the conservative treatment of early breast cancer. *International Journal of Surgical Investigation*.

[4] Singletary SE (2002). Surgical margins in patients with early-stage breast cancer treated with breast conservation therapy. *American Journal of Surgery*.

[5] Huston TL, Simmons RM (2005). Locally recurrent breast cancer after conservation therapy. *American Journal of Surgery*.

[6] DiBiase SJ, Komarnicky LT, Schwartz GF, Xie Y, Mansfield CM (1998). The number of positive margins influences the outcome of women treated with breast preservation for early stage breast carcinoma. *Cancer*.

[7] Vicini FA, Kestin L, Huang R, Martinez A (2003). Does local recurrence affect the rate of distant metastases and survival in patients with early-stage breast carcinoma treated with breast-conserving therapy?. *Cancer*.

[8] Jobsen JJ, Van Der Palen J, Ong F, Meerwaldt JH (2007). Differences in outcome for positive margins in a large cohort of breast cancer patients treated with breast-conserving therapy. *Acta Oncologica*.

[9] Scopa CD, Aroukatos P, Tsamandas AC, Aletra C (2006). Evaluation of margin status in lumpectomy specimens and residual breast carcinoma. *Breast Journal*.

[10] Fleming FJ, Hill ADK, Mc Dermott EW, O’Doherty A, O’Higgins NJ, Quinn CM (2004). Intraoperative margin assessment and re-excision rate in breast conserving surgery. *European Journal of Surgical Oncology*.

[11] McCormick JT, Keleher AJ, Tikhomirov VB, Budway RJ, Caushaj PF (2004). Analysis of the use of specimen mammography in breast conservation therapy. *American Journal of Surgery*.

[12] Chagpar A, Yen T, Sahin A (2003). Intraoperative margin assessment reduces reexcision rates in patients with ductal carcinoma in situ treated with breast-conserving surgery. *American Journal of Surgery*.

[13] Rubio IT, Mirza N, Sahin AA (2000). Role of specimen radiography in patients treated with skin-sparing mastectomy for ductal carcinoma in situ of the breast. *Annals of Surgical Oncology*.

[14] Ooi CWL, Serpell JW, Rodger A (2003). Tumour involvement of the re-excision specimen following clear local excision of breast cancer with positive margins. *ANZ Journal of Surgery*.

[15] Miller AR, Brandao G, Prihoda TJ, Hill C, Cruz AB, Yeh IT (2004). Positive/margins following surgical resection of breast carcinoma: analysis of pathologic correlates. *Journal of Surgical Oncology*.

[16] Papa MZ, Zippel D, Koller M, Klein E, Chetrit A, Ari GB (1999). Positive margins of breast biopsy: is reexcision always necessary?. *Journal of Surgical Oncology*.

[17] Assersohn L, Powles TJ, Ashley S (1999). Local relapse in primary breast cancer patients with unexcised positive surgical margins after lumpectomy, radiotherapy and chemoendocrine therapy. *Annals of Oncology*.

[18] Nasir N, Rainsbury RM (2003). The timing of surgery affects the detection of residual disease after wide local excision of breast carcinoma. *European Journal of Surgical Oncology*.

[19] Tot T (2005). DCIS, cytokeratins, and the theory of the sick lobe. *Virchows Archiv*.

